# Supposed Case of Poisoning by Copperas

**Published:** 1888-06

**Authors:** Pryor W. Fitts

**Affiliations:** Mountville, Ga.


### SUPPOSED CASE OF POISONING BY COPPERAS.

BY PRYOR W. FITTS, M. D., MOUNTVILLE, GA.

I report the following case of fatal poisoning for publication in THE JOURNAL, for the reason that such cases are of rare occurrence, so far as I can ascertain, yet the substance that did the fatal work is kept in almost every rural household.

About 12 o'clock on the night of February 24th, I was called in great haste to a colored child by its father, who informed me that it had been complaining for one or two days, and he, coming to the conclusion that it was affected with intestinal worms, had given it a dose of copperas, a substance that he had been in the habit of giving his children for that parasite. He further informed me that the sulphate of iron was given the child about five or six hours before, and that soon afterwards it vomited, and that very shortly subsequent to that, it retired and fell asleep.

Very soon after this the other members of the family retired and slept till just before I was summoned, when the father awoke, and, hearing the child making a rather strange noise, arose and examined it, and found it unable to speak.

On reaching the house, I found the child in its mother's arms. I learned that it was about five years of age, and, judging from the father's statement, I decided that about ten grains of the copperas had been given it. It paid no attention to those about it, and every one or two minutes very severe griping pains would come in its bowels. Its pulse was 110 and rather forcible. I produced vomiting, and the pains ceased. Mucilaginous drinks were given it afterwards. I left it quiet and doing well enough apparently. The next morning, however, I was called to it again, but on my arrival found it as quiet as I had left it the night previous, yet it made no effort to speak, nor paid any attention to what was occurring around it. Its pulse was 120, and not as forcible as it was the night before.

Learning that it had been constipated, a dose of castor oil was given it, and I requested its parents to continue the mucilaginous drinks.

It rested quietly till the oil operated, which was about 12 o'clock, when the griping pains returned in its bowels, and they increased in severity till it began to have convulsions, which lasted for several hours. I was summoned to it again, but did not reach it until after night on account of being from home, and one or two hours after the convulsions had ceased. On reaching its bed-side I found it in a collapsed condition: its pulse was imperceptible, respiration rapid, pupils widely dilated, and it was lying quietly, with the exception of an occasional groan. It died in a short time after my arrival.

Its bowels moved twice, and it vomited twice after the sulphate of iron was given. Its first vomit was soon after it took the iron. The first movement of its bowels, which was a small one, was a short time before I was first called to it. I produced its next vomit, also the next movement of its bowels. The case differs, so far as vomiting and purging are concerned, from cases that have been heretofore described who happened to the misfortune to get an overdose of this drug.

I desire to know, and I would be glad for some brother doctor to give me the desired information: was the child's death caused entirely from the intestinal irritation, or was it partly caused from absorption of the sulphate of iron?

